# New Variants of Nitroxide Mediated Polymerization

**DOI:** 10.3390/polym12071481

**Published:** 2020-07-02

**Authors:** Gérard Audran, Elena G. Bagryanskaya, Sylvain R. A. Marque, Pavel Postnikov

**Affiliations:** 1 Aix Marseille Univ, CNRS, ICR, UMR 7273, Case 551, Avenue Escadrille Normandie-Niemen, 13397 Marseille Cedex 20, France; 2 N. N. Vorozhtsov Novosibirsk Institute of Organic Chemistry Siberian Branch of Russian Academy of Sciences, Pr. Lavrentjeva 9, Novosibirsk 630090, Russia; 3 Novosibirsk State University, Pirogova str. 2, Novosibirsk 630090, Russia; 4 Department of Solid State Engineering, University of Chemistry and Technology, 16628 Prague, Czech Republic; 5 Research School of Chemistry and Applied Biomedical Sciences, Tomsk Polytechnic University, Lenin Ave, 30, Tomsk, Tomsk Oblast 634050, Russia

**Keywords:** nitroxide mediated polymerization, CI-NMP, SLNMP, PI-NMP, NMP2, ESCP

## Abstract

Nitroxide-mediated polymerization is now a mature technique, at 35 years of age. During this time, several variants have been developed: enhanced spin capture polymerization (ESCP), photoNMP (NMP2), chemically initiated NMP (CI-NMP), spin label NMP (SL-NMP), and plasmon-initiated NMP (PI-NMP). This mini-review is devoted to the features and applications of these variants.

## 1. Introduction

**Figure 1:**
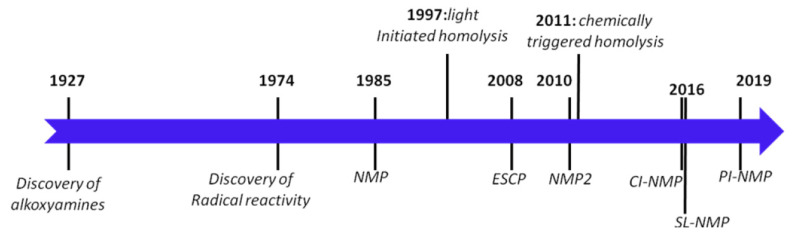
Timeline of the discoveries of the variants of NMP

**Scheme 1:**
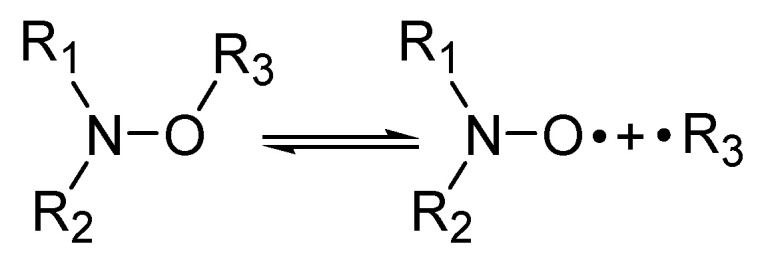
Reversible C—ON bond homolysis in alkoxyamines.

The first report on alkoxyamines was published by Jones and Major in 1927 (Figure 1) [1]. Nonetheless, it took almost half a century before Kovtun et al. [2] noticed the reversible thermal homolysis of alkoxyamines into alkyl and nitroxyl radicals (Scheme 1).

**Scheme 2:**
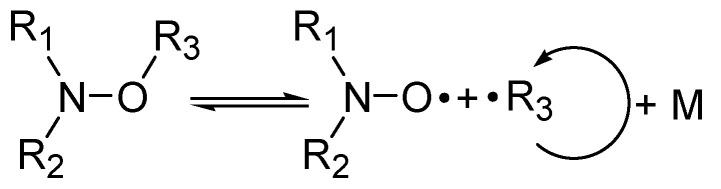
Oversimplified scheme of nitroxide-mediated polymerization (NMP) [4].

The first valuable application of alkoxyamines was reported by Rizzardo and coll. [3] when they discovered nitroxide-mediated polymerization in 1986 (Scheme 2) [4]. Indeed, they used reversible homolysis in the presence of monomers, and showed that a controlled insertion of units occurred, affording polymer chains of controlled sizes with re-activable ends. A decade later, this generated [5,6,7,8] a tremendous amount of investigations on the synthesis of initiators/controllers [9,10], the fundamental kinetics [11,12,13,14,15], the development of polymerization procedures [16], and the preparation of new materials [17,18,19,20,21].

All these investigations led to the development of variants of NMP. The variants discussed hereafter report non-conventional procedures for NMP (enhanced spin capture polymerization, aka ESCP, and spin labelled-NMP, aka SL-NMP), non-thermal initiation (photoNMP, aka NMP2 and plasmon initiated-NMP, aka PI-NMP), and non-conventional initiation (chemically initiated NMP, aka CI-NMP). In situ NMP corresponds to the in situ generation of alkoxyamines, unlike the conventional NMP, for which previously prepared alkoxyamines are used, and this has been thoroughly reviewed by Detrembleur and coll. [22] and Grishin et al. [23]. Surface initiated NMP (SI-NMP) is not considered, as it relies on the covalent attachment of siloxane-modified alkoxyamines onto a surface, and conventional NMP being performed next [24]. NMP performed in dispersed media has been discussed [25,26,27].

Interestingly, all the variants of NMP have been discovered in the last decade (Figure 1), and all the fundamental kinetics and procedures of conventional NMP have been laid out.

## 2. Generality in NMP 

**Scheme 3:**
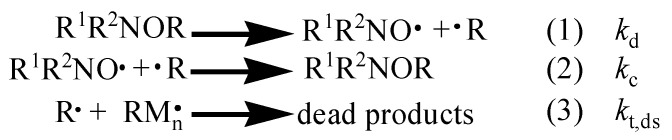
Elementary reactions involved in the alkoxyamine decomposition.

**Scheme 4:**
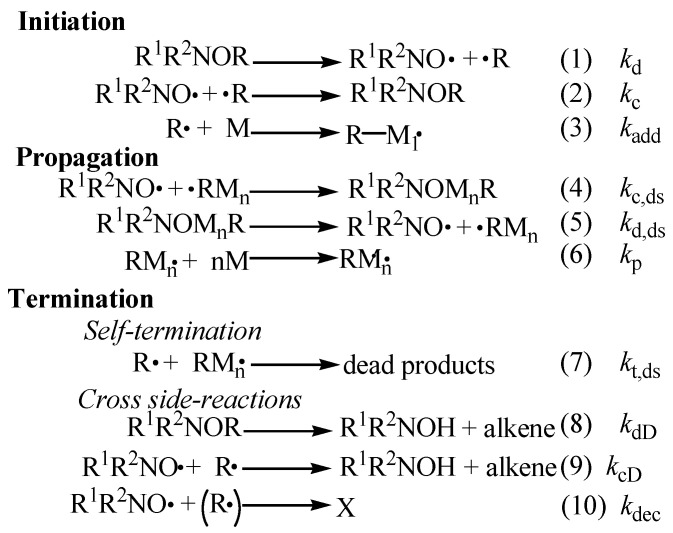
Kinetics of NMP: *k*_d_ is the rate constant for the homolysis of initiator, *k*_c_ for the re-formation of initiator, *k*_add_ for the addition of initiating alkyl radical onto monomer, *k*_c,ds_ for the re-formation reaction of dormant species (ds = macro-initiator), *k*_d,ds_ for the homolysis of dormant species, *k*_p_ for the propagation of the polymer chain, *k*_t,ds_ for the self-termination reactions of the propagating radicals, *k*_dD_ for the side-reactions occurring during the homolysis, *k*_cD_ for the side-reactions occurring during the re-formation reaction, and *k*_dec_ for the decay of nitroxide.

**Figure 2:**
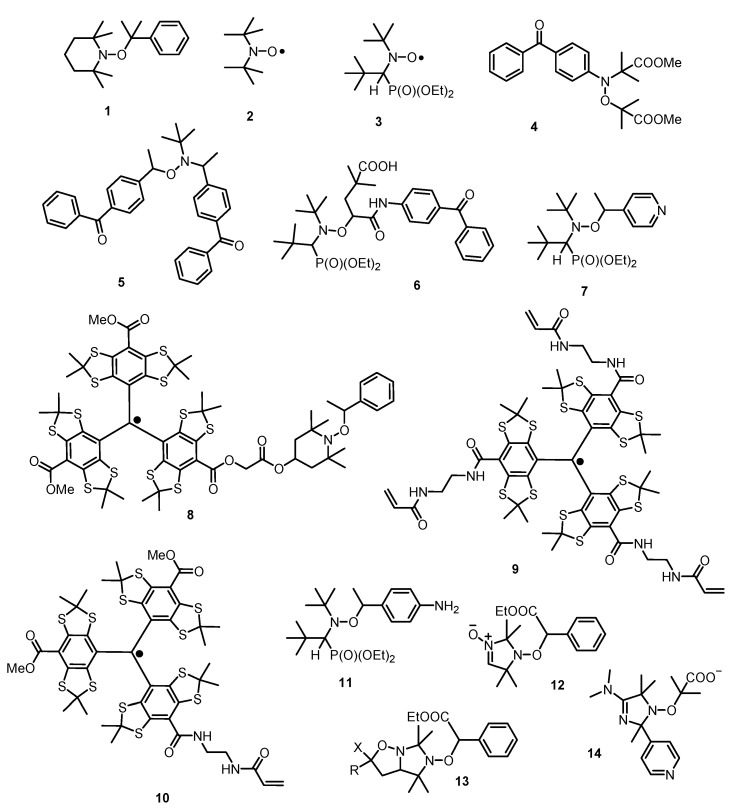
Alkoxyamines used to exemplify the variants of NMP

In their preliminary results, Rizzardo et al. [3,28] showed that NMP relies on the homolysis of the alkoxyamine C—ON bond and that the kinetics of polymerization can be modeled [29]. NMP relies [30] on the persistent radical effect (PRE) [31], a kinetic concept which was developed by Fischer [32] at the same time as but independently from NMP. In 1998, Fischer and coll. [33] showed that the evolution of species involved in the pseudo equilibrium displayed in Scheme 1 is controlled by the PRE, that is, the self-termination reaction of alkyl radical R_3_•, generated by the homolysis of alkoxyamine (dormant species) R_1_R_2_NOR_3_, affords an excess of nitroxide R_1_R_2_NO•, the persistent species, which favors the re-formation of alkoxyamine via the cross-coupling reaction with the alkyl radical R_3_•. The main consequence of the kinetics of Scheme 3 is an unexpectedly long lifetime for the alkoxyamines; that is, with alkoxyamine model **1** (Figure 2), at 83 °C, 2% decomposition is observed after 30000 s, whereas in the presence of radical scavengers, 100% decomposition is observed after 3000 s [4,33]. A few years later, it was shown that the equations [6,30,34,35] based on the kinetics of polymerization described in Scheme 4 hold for the polymerization of styrene using the nitroxides **2** [36] and **3** [37] as controlling agents. More details on kinetics as well as on the applications are available in the reviews listed in the reference section [4,5,6,7,8,9,10,11,12,15,16,17,18,19,20,21,22,23,24,25,26,27,30,38,39,40]. Interestingly, except for ESCP, the general comments on the kinetics of NMP hold for other variants.

## 3. Enhanced Spin Capture Polymerization ESCP

**Figure 3:**
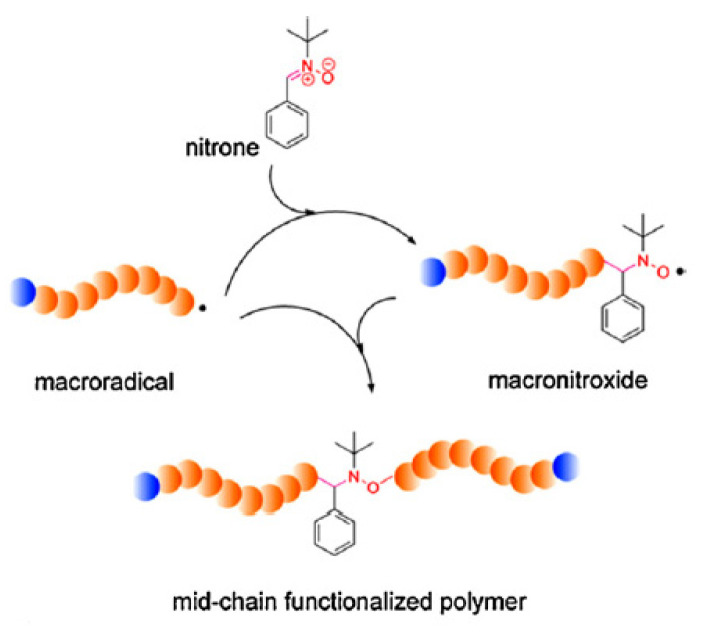
Scheme of electron spin capture polymerization (ESCP). Reprinted with the permission of Royal Society of Chemistry (RSC) from ref. [43].

This process has been promoted by Barner-Kowollik and coll. since 2008 [41,42,43,44,45]. Indeed, radical polymerization is initiated using a conventional initiator, then the growing polymeric chain radical is captured by a nitrone to generate a nitroxide. The latter is thus able to capture another polymeric chain radical to generate an alkoxyamine, affording a mid-chain functionalized polymer (Figure 3). When such a polymerization is performed at temperatures below 50 °C, the cross-coupling reaction between the alkyl and nitroxyl radicals is often considered as non-reversible (depending on the structure of the nitroxyl fragment) [45]. On the other hand, depending on the monomer [46], i.e., the polymer chain, and on the structure of the nitroxyl fragment, the alkoxyamine may behave as expected in conventional NMP, affording ABA triblock-copolymers in a straightforward way. ESCP is a valuable technique for readily reaching polymers exhibiting elaborate structures, such as miktoarm star polymers [47].

The numerical calculations on the kinetics of ESCP performed by Nikitin et al. [48] revealed the influence of different factors, in particular, the rate of initiation, spin capturing, including spin capturing rate constant and nitrone concentration, on the kinetics of the process and the characteristics of the polymer. Interestingly, the fast initiation of polymerization in ESCP can lead to the decrease in “living” chains that is absolutely contrary to NMP, where the rapid decomposition of the initiator is required for reaching the controlled regime. In the absence of side-reactions, the criteria for successful ESCP based on *k*_in_, *k*_p_, *k*_t_ and *C*_sc_ and initial concentrations of monomer and nitrone, allowing the prediction of molecular weight and DPn, were developed in the same way as was done for BMP by Fischer. As well as in NMP, side reactions have a detrimental effect on the kinetics of ESCP and the parameters of the formed polymer: an increase in the impact of the H-atom transfer leads to the broadening of the molecular weight distribution and a decrease in the “living” fraction. The other side-reaction, e.g., the homolysis of the C–ON bond, turns ESCP into NMP, increasing conversion and DP_n_ and decreasing Ð, but also reducing the number of “living” chains.

## 4. Nitroxide Mediated PhotoPolymerization NMP2

**Figure 4:**
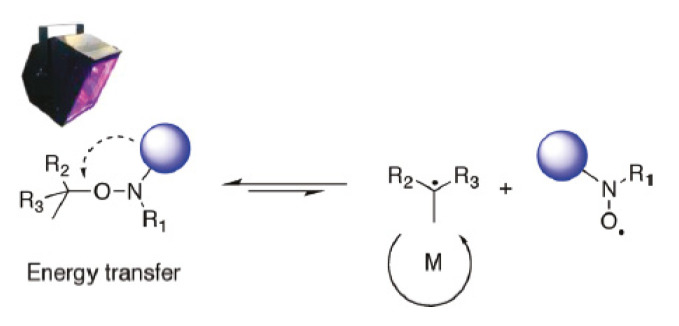
NMP2 concept. Reprinted with permission from (Guillaneuf, Y.; Bertin, D.; Gigmes, D.; Versace, D.-L.; Lalevée, J.; Fouassier, J.P. Toward Nitroxide-Mediated Photopolymerization. *Macromolecules* **2010**, *43*, 2204–2212). Copyright (2010) American Chemical Society.

Preliminary results on the homolysis of the C—ON bond under light irradiation were obtained with alkoxyamine **1** by Scaiano et al. in 1997 [49]. However, the system was not very efficient and did not raise too much interest. It was in 2008 that Yoshida and coll. [50,51] proposed first light-initiated NMP and in 2010 that Gigmes and coll. [52,53] proposed an efficient system based on alkoxyamine **4** as an example (Figure 4). 

**Figure 5:**
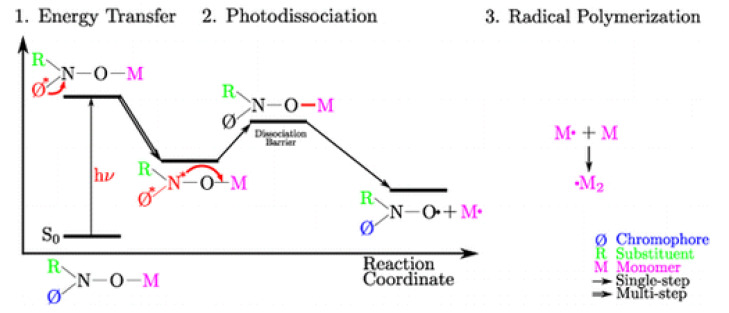
General scheme of the photochemically induced NMP2 initiation mechanism. The red part of the structure indicates the localization of the excitation energy in the structure. Reprinted with permission from (Huix-Rotllant, M.; Ferré, N. Theoretical Study of the Photochemical Initiation in Nitroxide-Mediated Photopolymerization. *J. Phys. Chem. A* **2014**, *118*, 4464–4470). Copyright (2014) American Chemical Society.".

Fundamental investigations revealed that the homolysis rate constants depend on the alkoxyamine structure, dye, wavelength, the power of the light and the attachment of the dye onto the nitroxyl fragment rather than onto the alkyl fragment. Density Functional Theory (DFT) calculations showed that the homolysis requires three steps (Figure 5): (i) light absorption and subsequent singlet-to-triplet conversion localized on the moiety carrying the dye; (ii) triplet energy transfer, in which the excitation energy is delocalized over the alkoxyamine moiety; and (iii) cleavage of the alkoxyamine O−C bond [54].

**Figure 6:**
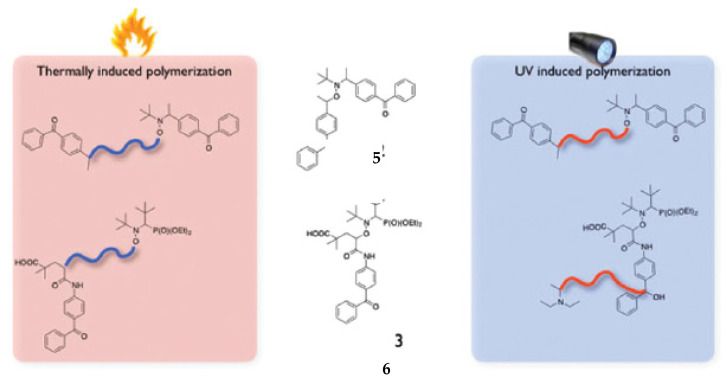
The two different polymerization modes that could be used with **5** and **6**. An amine is used as co-initiator for UV-induced polymerization with **6**. Reprinted with the permission of RSC from ref. [56]

NMP2 can be applied either in bulk [55] or for SI-NMP [24]. In the latter case, the best efficiency is observed when the alkyl fragment is attached to a surface and the dye to the nitroxyl fragment. Recently, the combination of UV-light-initiated NMP (NMP2) and thermal (conventional) NMP has been proposed to extend the range of applications of this variant (Figure 6) [56].

**Figure 7:**
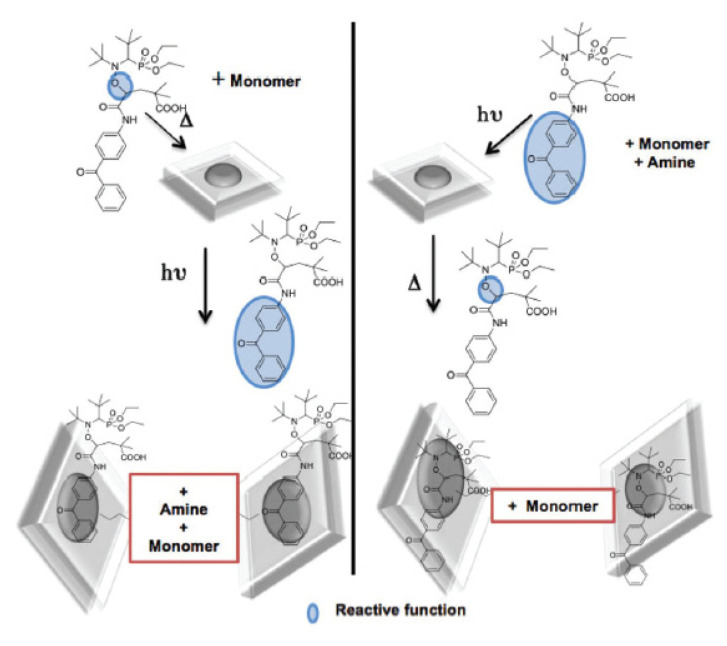
Use of a dual photoinitiator for the linkage of two polymer films by combining UV and thermal polymerization in laminate. Reprinted with the permission of RSC from ref. [56]

NMP2 is now an efficient approach for lithography [57]. It has also found many applications in materials science (Figure 7) [55,56].

## 5. Chemically Initiated Nitroxide Mediated Polymerization CI-NMP

**Figure 8:**
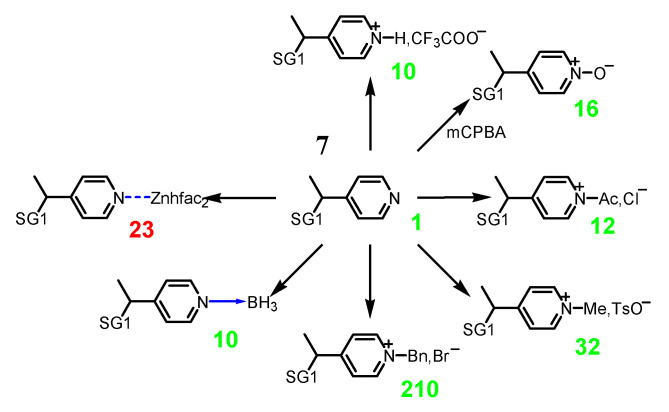
Various modes of activation of **7**. Bold (green for normal activation, red values for unexpected values for the coordination by Lewis acid) numbers are for relative *k*_d_ values.

A few years ago, the chemical activation by protonation of the alkyl fragment of alkoxyamine **7** was reported, with a 10-fold increase in *k*_d_ [58]. During the same period, Bagyanskaya and coll. [59] reported activation/de-activation events by the protonation of the nitroxyl fragment of alkoxyamine **14**. Later on, several modes of activation—oxidation [60], alkylation [60], acetylation [60], and coordination [61]—were investigated with **7**, affording very different responses (Figure 8) and providing a 23-fold increase in *k*_d_ for coordination with di(hexafluoroacetylacetonate) copper [61] and zinc ligands [62].

**Figure 9:**
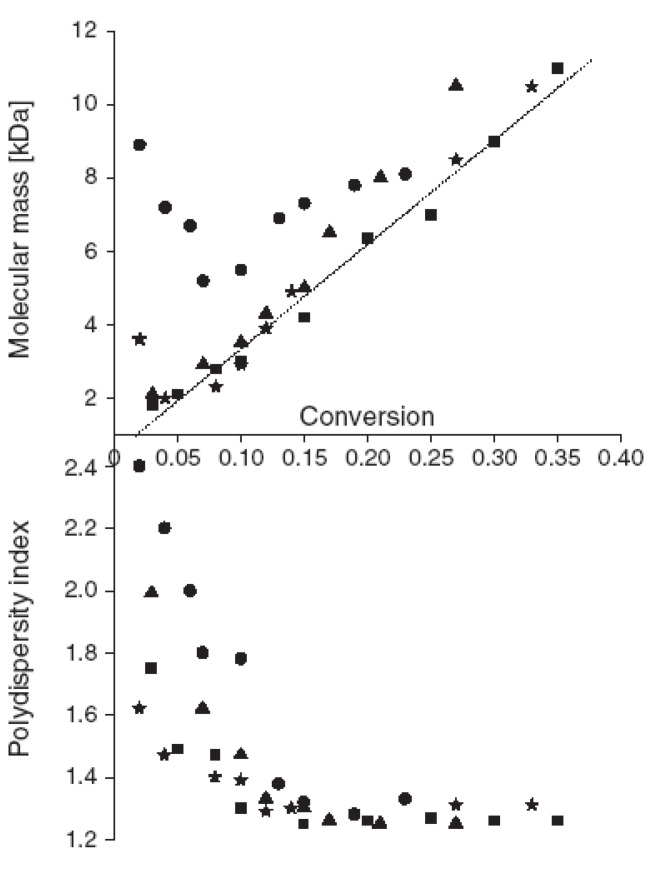
Plots of Mn versus conversion, Ð versus conversion, and ln([M]/[M0]) versus time for the polymerization of styrene at 90 °C initiated with *RS/SR*-**7** (●); *RS/SR*-**7Zn(hfac)_2_** (■); *in situ RS/SR*-**7Zn(hfac)_2_** (*RS/SR*-**7** + 0.5 equiv. Zn(hfac)_2_) (★); *RS/SR*-**7** + 10 equiv. Zn(hfac)_2_ (▲), the monomer-to-initiator ratio is 250 : 1. Reprinted with the permission of CSIRO from ref. [65]

Preliminary results on CI-NMP were obtained by protonation/deprotonation events [59,63] of **14**, which were applied to the NMP of styrene and acrylamide monomers. Later, NMP promoted by the protonation of the alkyl fragment of **7** was reported and exhibited the expected output from conventional NMP experiments [64]. CI-NMP was then developed using di(hexafluoroacetylacetonate) zinc ligand with **7** and showed an improvement in NMP features—linearity of Mn vs conversion and Ð vs conversion (Figure 9)—when low polymerization temperatures were applied [65].

The interest in CI-NMP relies on its application for SI-NMP. Indeed, activation would only occur for surface-coordinated initiators. Moreover, this would afford the opportunity for coordination and de-coordination at will for applications in self-healing polymers. 

**Figure 10:**
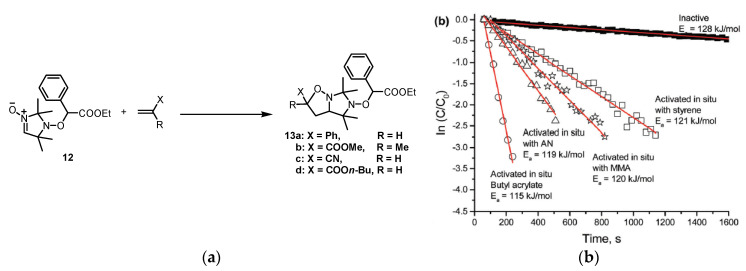
(**a**) Huysgen reaction between **12** and various alkenes; (**b**) *E*_a_ for activation in situ by the Huysgen reaction using various alkenes. Reprinted with permission from ref. [66]. Copyright 2019 Royal Society of Chemistry.

Another interest-arousing approach to the in situ initiation of alkoxyamine homolysis was recently proposed by Edeleva et al. [66]. This concept is based on the well-known fact that vinyl monomers involved in NMP usually show high reactivity in cycloaddition reactions, such as 1,3-dipolar cycloaddition (Figure 10). At room temperature, alkoxyamine **12** is relatively stable, thanks to the electron-withdrawing effect of the nitrone group and is ineffective as an NMP initiator. Under NMP conditions, if the substituent at the fourth position on the imidazoline ring is hydrogen, then it readily reacts with styrene, acrylonitrile, or acrylates to form tricyclic adducts such as **13a**, which have a much higher propensity for C–ON bond homolysis. The experimental kinetics measurements reveal the difference between the activation energy of alkoxyamine homolysis in the non-activated and activated state ~9 kJ/mol for styrene and 13 kJ/mol for butyl acrylate. 

Alkoxyamine **12** was used as model of safe initiators, i.e., switch from stable (safe molecule) to labile alkoxyamine (hazardous molecules), for NMP of styrene [66]. Moreover, by varying the alkyl fragment R in **13**—R can be a polymer chain, a bio-conjugate, a drug and so on—“click chemistry” is used both for the initiation of NMP using safer initiators for industry and for orthogonal conjugation.

## 6. Spin Labeled Nitroxide Mediated Polymerization SL-NMP

**Figure 11:**
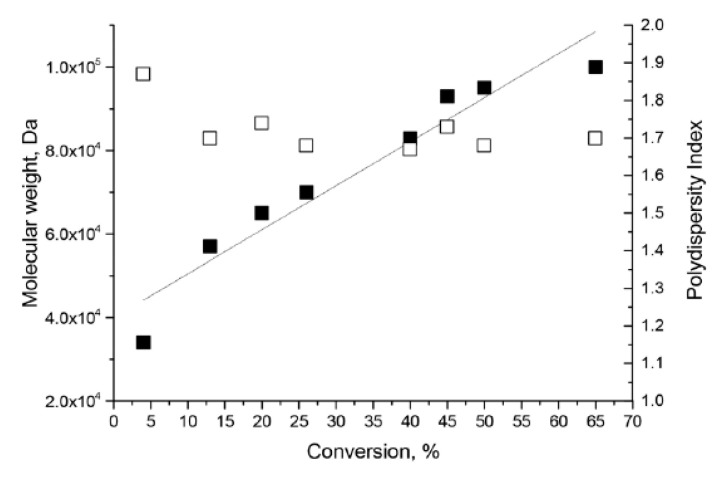
Molecular weight (left axis, filled squares) and polydispersity index (right axis, open squares) evolution vs. conversion plot for bulk polymerization of styrene initiated with alkoxyamine **8** at 130 °C with styrene/**8** ratio 5000:1. Dotted line for the theoretical evolution of Mn. Audran, G.; Bagryanskaya, E.; Bagryanskaya, I.; Brémond, P.; Edeleva, M.; Marque, S.R.A.; Parkhomenko, D.; Rogozhnikova, O.Yu.;. Tormyshev, V.M.; Tretyakov, E.V.; Trukhin, D.V.; Zhivetyeva, S. Trityl-based Alkoxyamines as NMP Controlers and Spin-labels. *Polym. Chem.* **2016**, *7*, 6490–6499. Published by The Royal Society of Chemistry.

Polymerization-initiated self-assembly (PISA) materials have been a field of interest for several years [26]. Noteworthily, for the investigation of the dynamics of protein, of RNA/DNA, of protein–protein interactions, the use of the EPR and site-directed spin labeling (SDSL) method has been well developed for several decades [67]. On the other hand, the investigation of the nanostructuration of polymers/materials using these methods is of increasing interest [68,69,70]. Indeed, the most convenient technique to label end-chain polymers is the use of “click” reactions. However, with such an approach, whose efficiency depends on the organization of the materials, the labeling is lower than 100%. This might make investigations quite challenging, and it impedes the development of this field. Although performing controlled radical polymerization in the presence of two different types of persistent radicals seems at first glance to be strange, we have decided to investigate this approach. Prequels [71,72,73,74] on controlled radical polymerization have reported the use of trityl radical as a controlling agent with very moderate success. Taking into account this observation, we developed SL-NMP with the trityl radical moiety attached either onto the nitroxyl fragment or onto the alkyl fragment, as exemplified with **8** [75], to ensure a successful NMP experiment with two persistent radicals: a nitroxide as controller and a trityl radical as a spectator. As displayed in Figure 11, a good control was observed, as highlighted by the linear plot of Mn vs conversion and by values of Ð close to 1.5. Moreover, after the purification of the polymer, more than 90% livingness was observed, as usual, and more than 90% of trityl radical was recovered. Obviously, controlled radical polymerization such as NMP can be performed in the presence of another type of persistent radical whatever its position: on the alkyl fragment for -end chain labeled polymers or on the nitroxyl fragment for -end chain labeled polymers. 

**Figure 12:**
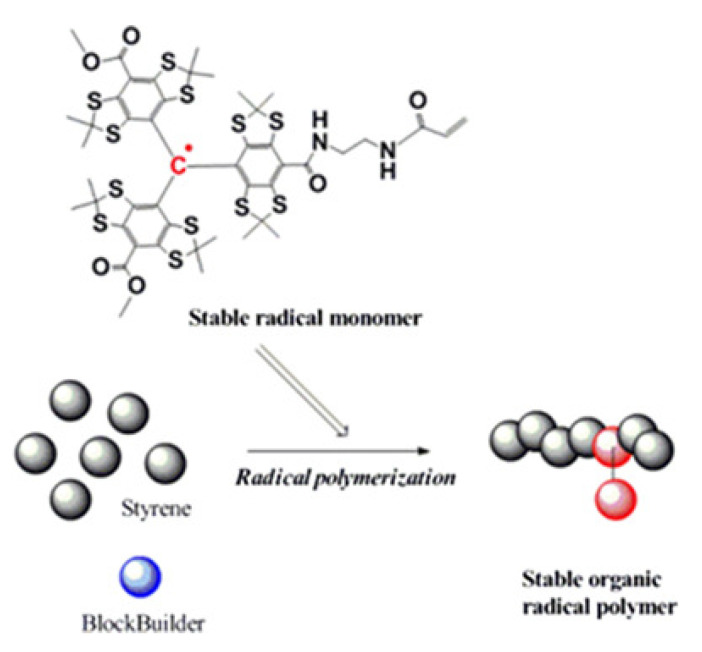
Preparation of stable organic radical polymer (SORP) using conventional radical polymerization and NMP. Reprinted with the permission of Wiley from ref. [78].

With these results in hand, we turned our interest to the preparation of a stable organic radical polymer (SORP), i.e., a polymer backbone made up of monomer units carrying a persistent radical such as a nitroxide. Indeed, in the literature, it was quoted [76,77]: “free-radical polymerization techniques cannot be used in the direct polymerization method” which is in sharp contrast with our claims concerning SL-NMP. Therefore, two types of trityl monomers were prepared—a trityl radical **9** carrying three acrylamides as monomer units and a trityl radical **10** carrying one unit of acrylamide as monomer—and were investigated in conventional polymerization and in NMP (Figure 12) [78]. Depending on the experimental conditions, up to 90% of trityl radicals were recovered both in crude and purified materials. These results strikingly highlight the potential of SL-NMP for the preparation of SORP. 

## 7. Plasmon Initiated Nitroxide Mediated Polymerization PI-NMP

A decade ago, plasmon-induced chemistry emerged, and has since raised a keen interest [79,80]. The plasmon effect has found many applications in various fields, such as sensors [81], nanomaterials [82], optics [83], biology [84], data storage [85], and so on. Nevertheless, nowadays scientists are paying great attention to the influence of plasmon on the reaction rate in the transformation of organic molecules on the surfaces of plasmon-active nanomaterials [86,87]. The exact mechanism of the interaction of plasmon with organic molecules is still questionable and has been discussed extensively recent years [88,89]. Anyway, now we can certainly confirm that plasmonic energy significantly enhances the rate of the reaction and provides an alternative method for the activation of chemical transformations, such as Pd-catalyzed cross-coupling reactions [90,91], hydrogenations [92,93,94], cycloadditions [95,96], oxidative reactions [97,98].

The synthetic potential of plasmon-induced transformations led us towards its application to surface-assisted polymerization, which can provide a novel tool for the design of materials with controlled properties. Thus, a surface covered with a layer of polymer may dramatically change the properties of the material, provided that the composition and the size of the polymer layer are well defined and controlled. Moreover, such material must be both easily accessible and cheaply processed. Up to now, the most convenient approach has been to use surface-initiated controlled radical polymerization techniques. However, such an approach requires specific procedures to remove all initiators that are not attached to the surface. Obviously, in such a case the plasmon-induced polymerization can sufficiently improve the creation of the targeted materials. 

**Figure 13:**
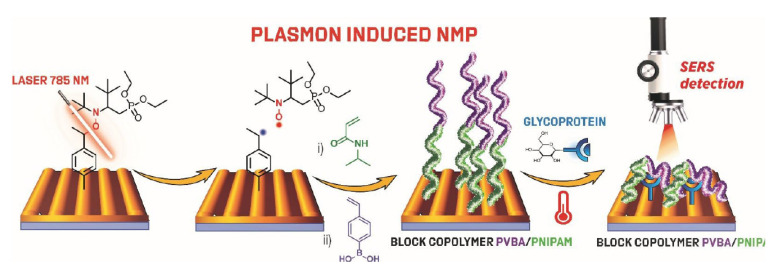
Scheme for plasmon-initiated NMP (PI-NMP). Reprinted with the permission of RSC from ref. [102].

The first application of plasmon energy for the polymerization was proposed by Baumberg [99,100], where styrene underwent radical polymerization between two nanoantennas. Slightly later, some attempts were made with RAFT agents [101]. At first glance, such an approach was not suitable for atom transfer radical polymerization. By contrast, the first report [102] of PI-NMP by our teams was successful, and highlighted the potential of the combination of the plasmon effect and NMP (Figure 13). In the first step, alkoxyamine **11** was attached onto the gold surface via diazonium chemistry. Then, upon laser irradiation at 785 nm, the homolysis of the C–ON bond of the grafted alkoxyamine occurred at room temperature, initiating radical polymerization of *N*-isopropyl acrylamide (NIPAM) and of styrene boronic acid (VBA). As the homolysis of the C–ON bond was controlled by the plasmon effect, it was possible to prepare a block co-polymer of 15 NIPAM units and 15 VBA units. This new material was applied to the detection of glycoproteins using surface enhanced Raman spectroscopy (SERS).

## 8. Conclusions

Two among the five new variants of NMP—ESCP and NMP2—are already commonly used for the preparation of new materials. The other three variants—CI–NMP, SL–NMP, and PI–NMP—have been developed more recently and exhibit a high potential: CI–NMP should find application in self-healing polymers by combining orthogonal reactivity (i.e., coordination/de-coordination and reversible homolysis), SL–NMP is interesting for the investigation of materials prepared using PISA techniques or for the preparation of SORP using radical polymerization of monomers carrying persistent radicals, and PI–NMP is highly valuable for the preparation of smart materials using metal- and UV-light-free polymerization at room temperature.

## References

[1] Jones L.W., Major R.T. (1927). Substituted O-alkyl hydroxylamines chemically related to medicinally valuable amines. J. Am. Chem. Soc..

[2] Kovtun G.A., Aleksandrov A.L., Golubev V.A. (1974). Interaction of Peroxide Radicals with Esters of Hydroxylamines. Russ. Chem. Bull..

[3] Solomon D.H., Rizzardo E., Cacioli P. (1986). Polymerization Process and Polymers Produced Thereby. U.S. Patent.

[4] Bertin D., Gigmes D., Marque S.R.A., Tordo P. (2011). Kinetic Subtleties of Nitroxide Mediated Polymerization. Chem. Soc. Rev..

[5] Matyjaszewski K. (1996). Controlled Radical Polymerization. Current Opin. Solid State Materials Sci..

[6] Bagryanskaya E.G., Marque S.R.A., Gigmes D. (2016). Kinetic Aspects of Nitroxide-Mediated Polymerization. RSC Polymer Chemistry Series, n°=19, Nitroxide Mediated Polymerization: From Fundamentals to Applications in Materials Sciences.

[7] Greszta D., Mardare D., Matyjaszewski K. (1994). “Living” Radical Polymerization. 1. Possibilities and Limitations. Macromolecules.

[8] Kreutzer J., Yagci Y. (2018). Metal Free Reversible-Deactivation Radical Polymerizations: Advances, Challenges, and Opportunities. Polymers.

[9] Grubbs R.B. (2011). Nitroxide-Mediated Radical Polymerization: Limitations and Versatility. Polymer Rev..

[10] Tebben L., Studer A. (2011). Nitroxides: Applications in Synthesis and in Polymer Chemistry. Angew. Chem. Int. Ed..

[11] Fischer H. (2001). The Persistent Radical Effect: A Principle for Selective Radical Reactions and Living Radical Polymerizations. Chem. Rev..

[12] Fischer H., Souaille M. (2001). The Persistent Radical Effect in Living Radical Polymerization - Borderline Cases and Side-Reactions. Macromol. Symp..

[13] Fortunatti C., Sarmoria C., Brandolin A., Asteasuain M. (2013). Theoretical Analysis of Nitroxide-Mediated Copolymerization of Styrene and A-Methyl-Styrene Under Different Operating Policies and Reactor Designs. Macromol. React. Engineer..

[14] Fukuda T., Goto A., Ohno K. (2000). Mechanisms and Kinetics of Living Radical Polymerizations. Macromol. Rapid Commun..

[15] Goto A., Fukuda T. (2004). Kinetics of Living Radical Polymerization. Progr. Polym. Sci..

[16] Braunecker W.A., Matyjaszewski K. (2007). Controlled/Living Radical Polymerization: Features, Developments, and Perspectives. Progr. Polym. Sci..

[17] Gao H., Matyjaszewski K. (2009). Synthesis of Functional Polymers with Controlled Architecture by CRP of Monomers in the Presence of Cross-Linkers: From Stars to Gels. Progr. Polym. Sci..

[18] Grishin D.F., Grishin I.D. (2012). Controlled Radical Polymerization: Prospects for Application for Industrial Synthesis of Polymers. Russ. J. Appl. Chem..

[19] Kermagoret A., Gigmes D. (2016). Combined Nitroxide Mediated Radical Polymerization Techniques for Block Copolymer Synthesis. Tetrahedron.

[20] Destarac M. (2018). Industrial Development of Reversible-Deactivation Radical Polymerization: Is the Induction Period Over?. Polym. Chem..

[21] Destarac M. (2010). Controlled Radical Polymerization: Industrial Stakes, Obstacles and Achievements. Macromol. React. Engineer..

[22] Sciannamea V., Jérôme R., Detrembleur C. (2008). In-Situ Nitroxide-Mediated Radical Polymerization (NMP) Processes: Their Understanding and Optimization. Chem. Rev..

[23] Kolyakina E.V., Grishin D.F. (2009). Nitroxide Radicals Formed in Situ as Polymer Chain Growth Regulators. Russ. Chem. Rev..

[24] Zoppe J.O., Ataman N.C., Mocny P., Wang J., Moraes J., Klok H.A. (2017). Surface-Initiated Controlled Radical Polymerization: State-of-the-Art, Opportunities, and Challenges in Surface and Interface Engineering with Polymer Brushes. Chem. Rev..

[25] Charleux B., Nicolas J. (2007). Water-Soluble SG1-Based Alkoxyamines: A Breakthrough in Controlled/Living Free-Radical Polymerization in Aqueous Dispersed Media. Polymer.

[26] Zetterlund P.B., Thickett S.C., Perrier S., Bourgeat-Lami E., Lansalot M. (2015). Controlled/Living Radical Polymerization in Dispersed Systems: An Update. Chem. Rev..

[27] Cunningham M.F. (2008). Controlled/Living Radical Polymerization in Aqueous Dispersed Systems. Progr. Polym. Sci..

[28] Moad G., Rizzardo E. (1995). Alkoxyamine-Initiated Living Radical Polymerization: Factors Affecting Alkoxyamine Homolysis Rates. Macromolecules.

[29] Johnson C., Moad G., Solomon D.H. (1990). The Application of Supercomputers in Modeling Chemical Reaction Kinetics: Kinetic Simulation of“Quasi-Living”Radical Polymerization. Aust. J. Chem..

[30] Gigmes D., Marque S.R.A., Chatgilialoglu C., Studer A. (2012). Nitroxide Mediated Polymerization and its Applications. Encyclopedia of Radicals in Chemistry, Biology, and Materials.

[31] This term has been coined by Daikh B.E., Finke R.G. (1992). The Persistent Radical Effect: A Prototype Example of Extreme, 10^5^ to 1, Product Selectivity in a Free-Radical Reaction Involving Persistent. Co^II^[Macrocycle] and Alkyl Free Radicals. J. Am. Chem. Soc..

[32] Fischer H. (1986). Unusual Selectivities of Radical Reactions by Internal Suppression of Fast Modes. J. Am. Chem. Soc..

[33] Kothe T., Marque S., Martschke R., Popov M., Fischer H. (1998). Radical Reaction Kinetics During Homolysis of N-alkoxyamines: Verification of the Persistent Radical Effect. J. Chem. Soc., Perkin Trans..

[34] Yoshikawa C., Goto A., Fukuda T. (2002). Quantitative Comparison of Theory and Experiment on Living Radical Polymerization Kinetics. 1. Nitroxide-Mediated Polymerization. Macromolecules.

[35] Tang W., Fukuda T., Matyjaszewski K. (2006). Reevaluation of Persistent Radical Effect in NMP. Macromolecules.

[36] Ohno K., Tsujii Y., Miyamoto T., Fukuda T., Goto M., Kobayashi K., Akaike T. (1998). Synthesis of a Well-Defined Glycopolymer by Nitroxide-Controlled Free Radical Polymerization. Macromolecules.

[37] Lutz J.F., Desmazes P.L. (2001). The Persistent Radical Effect in Nitroxide Mediated Polymerization: Experimental Validity. Macromol. Rapid Commun..

[38] Nicolas J., Guillaneuf Y., Lefay C., Bertin D., Gigmes D., Charleux B. (2013). Nitroxide-Mediated Polymerization. Progr. Polym. Sci..

[39] Garcia-Valdez O., Champagne P., Cunningham M.F. (2018). Graft Modification of Natural Polysaccharides via Reversible Deactivation Radical Polymerization. Progr. Polym. Sci..

[40] Darabi A., Jessop P.G., Cunningham M.F. (2016). CO_2_-Responsive Polymeric Materials: Synthesis, Self-Assembly, and Functional Applications. Chem. Soc. Rev..

[41] Wong E.H.H., Junkers T., Barner-Kowollik C. (2008). Enhanced Spin Capturing Polymerization: An Efficient and Versatile Protocol for Controlling Molecular Weight Distributions. J. Polym. Sci. A Polym. Chem..

[42] Wong E.H.H., Stenzel M.H., Junkers T., Barner-Kowollik C. (2009). The Kinetics of Enhanced Spin Capturing Polymerization: Influence of the Nitrone Structure. J. Polym. Sci. A Polym. Chem..

[43] Wong E.H.H., Boyer C., Stenzel M.H., Barner-Kowollik C., Junkers T. (2010). Spin Capturing with Nitrones: Radical Coupling Reactions with Concurrent Introduction of Mid-Chain Functionality. Chem. Commun..

[44] Junkers T., Wong E.H.H., Stenzel M.H., Barner-Kowollik C. (2009). Formation Efficiency of ABA Blockcopolymers via Enhanced Spin Capturing Polymerization (ESCP): Locating the Alkoxyamine Function. Macromolecules.

[45] Wong E.H.H., Junkers T., Barner-Kowollik C. (2011). Nitrones in Synthetic Polymer Chemistry. Polym. Chem..

[46] Dommanget C., Boisson C., Charleux B., D’Agosto F., Monteil V., Boisson F., Junkers T., Barner-Kowollik C., Guillaneuf Y., Gigmes D. (2013). Enhanced Spin Capturing Polymerization of Ethylene. Macromolecules.

[47] Wong E.H.H., Stenzel M.H., Junkers T., Barner-Kowollik C. (2010). Spin Capturing with “Clickable” Nitrones: Generation of Miktoarmed Star Polymers. Macromolecules.

[48] Nikitin S.V., Parkhomenko D.A., Edeleva M.V., Bagryanskaya E.G. (2015). Enhanced spin capturing polymerization: Numerical investigation of mechanism. J. Polym. Sci.: Part. A: Polym. Chem..

[49] Scaiano J.C., Connolly T.J., Mohtat N., Pliva C.N. (1997). Exploratory Study of the Quenching of Photosensitizers by Initiators of Free Radical “Living” Polymerization. Can. J. Chem..

[50] Yoshida E. (2008). Photo-Living Radical Polymerization of Methyl Methacrylate by a Nitroxide Mediator. Colloid. Polym. Sci..

[51] Yoshida E. (2010). Nitroxide-Mediated Photo-Living Radical Polymerization of Methyl Methacrylate in Solution. Colloid. Polym. Sci..

[52] Guillaneuf Y., Bertin D., Gigmes D., Versace D.-L., Lalevée J., Fouassier J.P. (2010). Toward Nitroxide-Mediated Photopolymerization. Macromolecules.

[53] Guillaneuf Y., Versace D.-L., Bertin D., Lalevée J., Gigmes D., Fouassier J.P. (2010). Importance of the Position of the Chromophore Group on the Dissociation Process of Light Sensitive Alkoxyamines. Macromol. Rapid Commun..

[54] Huix-Rotllant M., Ferré N. (2014). Theoretical Study of the Photochemical Initiation in Nitroxide-Mediated Photopolymerization. J. Phys. Chem. A.

[55] Pan X., Tasdelen M.A., Laun J., Junkers T., Yagci Y., Matyjaszewski K. (2016). Photomediated Controlled Radical Polymerization. Progr. Polym. Sci..

[56] Morris J., Telitel S., Fairfull-Smith K.E., Bottle S.E., Lalevée J., Clément J.-L., Guillaneuf Y., Gigmes D. (2015). Novel Polymer Synthesis Methodologies Using Combinations of Thermally- and Photochemically-Induced Nitroxide Mediated Polymerization. Polym. Chem..

[57] Garra P., Dietlin C., Morlet-Savary F., Dumur F., Gigmes D., Fouassier J.P., Lalevée J. (2017). Photopolymerization Processes of Thick Films and in Shadow Areas: A Review for the Access to Composites. Polym. Chem..

[58] Brémond P., Marque S.R.A. (2011). First Proton Triggered C—ON Bond Homolysis in Alkoxyamines. Chem. Commun..

[59] Edeleva M.V., Kirilyuk I.A., Zhurko I.F., Parkhomenko D.A., Tsentalovich Y.P., Bagryanskaya E.G. (2011). pH-Sensitive C–ON Bond Homolysis of Alkoxyamines of Imidazoline Series with Multiple Ionizable Groups as an Approach for Control of Nitroxide Mediated Polymerization. J. Org. Chem..

[60] Brémond P., Koïta A., Marque S.R.A., Pesce V., Roubaud V., Siri D. (2012). Chemically Trigerred C—ON Bond Homolysis of Alkoxyamines. Quaternization of the Alkyl Fragments. Org. Lett..

[61] Audran G., Bagryanskaya E., Bagryanskaya I., Brémond P., Edeleva M., Marque S.R.A., Parkhomenko D., Tretyakov E., Zhivetyeva S. (2016). C—ON Bond Homolysis of Alkoxyamines Triggered by Paramagnetic Copper(II) Salts. Inorg. Chem. Frontier.

[62] Audran G., Bagryanskaya E., Bagryanskaya I., Edeleva M., Marque S.R.A., Parkhomenko D., Tretyakov E., Zhivetyeva S. (2017). Zinc(II) Hexafluoroacetylacetonate Complexes of Alkoxyamines: NMR and Kinetic Investigations. First Step for a New Way to Prepare Hybrid Materials. ChemistrySelect.

[63] Edeleva M.V., Bagryanskaya E.G., Marque S.R.A. (2018). Imidazoline and Imidazolidine Nitroxides as Controlling Agents in Nitroxide-Mediated Pseudo-living Radical Polymerization. Russ. Chem. Rev..

[64] Bagryanskaya E., Brémond P., Edeleva M., Marque S.R.A., Parkhomenko D., Roubaud V., Siri D. (2012). Chemically Triggered C—ON Bond Homolysis in Alkoxyamines. Part 2: DFT Investigation and Application of the pH Effect on NMP. Macromol. Rapid Commun..

[65] Audran G., Bagryanskaya E., Edeleva M., Marque S.R.A., Parkhomenko D., Tretyakov E., Zhivetyeva S. (2018). Coordination-Initiated Nitroxide-Mediated Polymerization (CI-NMP). Aust. J. Chem..

[66] Edeleva M., Morozov D., Parkhomenko D., Polienko Y., Iurchenkova A., Kirilyuk I., Bagryanskaya E. (2019). Versatile approach to activation of alkoxyamine homolysis by 1, 3-dipolar cycloaddition for efficient and safe nitroxide mediated polymerization. Chemical Communications.

[67] Bagryanskaya E.G., Krumkacheva O., Fedin M.V., Marque S.R.A. (2015). Development and Application of Spin Traps, Spin Probes, and Spin Labels. Methods Enzym..

[68] Gentilini C., Franchi P., Mileo E., Polizzi S., Lucarini M., Pasquato L. (2009). Formation of Patches on 3D SAMs Driven by Thiols with Immiscible Chains Observed by ESR Spectroscopy. Angew. Chem. Int. Ed..

[69] Ong Q., Luo Z., Stellacci F. (2017). Characterization of Ligand Shell for Mixed-Ligand Coated Gold Nanoparticles. Acc. Chem. Res..

[70] Naveed K.-U.-R., Wang L., Yu H., Ullah R.S., Haroon M., Fahad S., Li J., Elshaarani T., Khan R.U., Nazir A. (2018). Recent Progress in the Electron Paramagnetic Resonance Study of Polymers. Polym. Chem..

[71] Ebdon J.R., Huckerby T.N., Hunt B.J., Rimmer S. (1998). Radical Polymerizations of Methyl Methacrylate Initiated by Methyl 2-[(4-Diphenylmethylene)-2, 5-Cyclohexadienyl]-2-Methyl-Propanoate: A Model System for So-Called “quasi-living” polymerization of methyl methacrylate initiated by phenylazotriphenylmethane. Polymer.

[72] Otsu T., Yoshida M., Tazaki T. (1982). A Model for Living Radical Polymerization. Makromol. Chem. Rapid Commun..

[73] Acar M.H., Yagci Y. (1991). Studies on the Block Copolymerization of Methacrylo-Nitrile and Hexafluorobutylmethacrlate Using Phenylazo-Triphenylmethane as Thermal Iniferter. J. Macromol. Sci.: Part. A – Chem..

[74] Chernikova E.V., Garina E.S., Zeremskii M.Y., Olenin A.V., Lachinov M.B., Golubev V.B. (1995). Quasiliving radical polymerization of methyl methacrylate in the presence of phenylazotriphenylmethane. Polym. Sci. Ser. A..

[75] Audran G., Bagryanskaya E., Bagryanskaya I., Brémond P., Edeleva M., Marque S.R.A., Parkhomenko D., Rogozhnikova O.Y., Tormyshev V.M., Tretyakov E.V. (2016). Trityl-based Alkoxyamines as NMP Controlers and Spin-labels. Polym. Chem..

[76] Zhang K., Monteiro M.J., Jia Z. (2016). Stable Organic Radical Polymers: Synthesis and Applications. Polym. Chem..

[77] Hansen K.-A., Blinco J.P. (2018). Nitroxide Radical Polymers – a Versatile Material Class for High-Tech Applications. Polym. Chem..

[78] Edeleva M.V., Marque S.R.A., Rogozhnikova O.Y., Tormyshev V.M., Troitskaya T.I., Bagryanskaya E.G. (2018). Radical Polymerization of Radical-labelled Monomers: The Triarylmethyl-based Radical Monomer as an Example. J. Polym. Sci.: Part. A: Polym. Chem..

[B79-polymers-12-01481] 79.See Accounts of Chemical Research special issue “Nanochemistry for Plasmonics and Plasmonics for Nanochemistry”.

[80] Nam J.-M., Liz-Marzán L., Halas N. (2019). Chemical Nanoplasmonics: Emerging Interdisciplinary Research Field at Crossroads Between Nanoscale Chemistry and Plasmonics. Acc. Chem. Res..

[81] Wang P., Nasir M.E., Krasavin A.V., Dickson W., Jiang Y., Zayats A.V. (2019). Plasmonic Metamaterials for Nanochemistry and Sensing. Acc. Chem. Res..

[82] Zhan C., Chen X.-J., Huang Y.-F., Wu D.-Y., Tian Z.-Q. (2019). Plasmon-Mediated Chemical Reactions on Nanostructures Unveiled by Surface-Enhanced Raman Spectroscopy. Acc. Chem. Res..

[83] Gargiulo J., Berté R., Li Y., Maier S.A., Cortés E. (2019). From Optical to Chemical Hot Spots in Plasmonics. Acc. Chem. Res..

[84] Murphy C.J., Chang H.-H., Falagan-Lotsch P., Gole M.T., Hofmann D.M., Hoang K.N.L., McClain S.M., Meyer S.M., Turner J.G., Unnikrishnan M. (2019). Virus-Sized Gold Nanorods: Plasmonic Particles for Biology. Acc. Chem. Res..

[85] Phan-Quang G.C., Han X., Koh C.S.L., Sim H.Y.F., Lay C.L., Leong S.X., Lee Y.H., Pazos-Perez N., Alvarez-Puebla R.A., Ling X.Y. (2019). Three-Dimensional Surface-Enhanced Raman Scattering Platforms: Large-Scale Plasmonic Hotspots for New Applications in Sensing, Microreaction, and Data Storage. Acc. Chem. Res..

[86] Zhang X., Yao X.K.J. (2018). Recent development of plasmon-mediated photocatalysts and their potential in selectivity regulation. J. Mater. Chem. A.

[87] Zhang Y., He S., Guo W., Hu Y., Huang J., Mulcahy J.R., Wei W.D. (2018). Surface-Plasmon-Driven Hot Electron Photochemistry. Chem. Rev..

[88] Kazuma E., Kim Y. (2019). Mechanistic Studies of Plasmon Chemistry on Metal Catalysts. Angew. Chem. Int. Ed..

[89] Zhan C., Chen X.-J., Yi J., Li J.-F., Wu D.-Y., Tian Z.-Q. (2018). From plasmon-enhanced molecular spectroscopy to plasmon-mediated chemical reactions. Nat. Rev. Chem..

[90] Wang F., Li C., Chen H., Jiang R., Sun L.-D., Li Q., Wang J., Yu J.C., Yan C.-H. (2013). Plasmonic Harvesting of Light Energy for Suzuki Coupling Reactions. J. Am. Chem. Soc..

[91] Xiao Q., Sarina S., Bo A., Jia J., Liu H., Arnold D.P., Huang Y., Wu H., Zhu H. (2014). Visible Light-Driven Cross-Coupling Reactions at Lower Temperatures Using a Photocatalyst of Palladium and Gold Alloy Nanoparticles. ACS Catalysis.

[92] Guselnikova O., Olshtrem A., Kalachyova Y., Panov I., Postnikov P., Svorcik V., Lyutakov O. (2018). Plasmon Catalysis on Bimetallic Surface—Selective Hydrogenation of Alkynes to Alkanes or Alkenes. The J. Phys. Chem. C.

[93] Landry M.J., Gellé A., Meng B.Y., Barrett C.J., Moores A. (2017). Surface-Plasmon-Mediated Hydrogenation of Carbonyls Catalyzed by Silver Nanocubes under Visible Light. ACS Catalysis.

[94] Yin Z., Wang Y., Song C., Zheng L., Ma N., Liu X., Li S., Lin L., Li M., Xu Y. (2018). Hybrid Au–Ag Nanostructures for Enhanced Plasmon-Driven Catalytic Selective Hydrogenation through Visible Light Irradiation and Surface-Enhanced Raman Scattering. J. Am. Chem. Soc..

[95] Chuang C.-C., Chu H.-C., Huang S.-B., Chang W.-S., Tuan H.-Y. (2020). Laser-induced plasmonic heating in copper nanowire fabric as a photothermal catalytic reactor. Chem. Engineer. J..

[96] Guselnikova O., Postnikov P., Chehimi M.M., Kalachyovaa Y., Svorcik V., Lyutakov O. (2019). Surface Plasmon-Polariton: A Novel Way To Initiate Azide–Alkyne Cycloaddition. Langmuir.

[97] Xiao Q., Connell T.U., Cadusch J.J., Roberts A., Chesman A.S.R., Gómez D.E. (2018). Hot-Carrier Organic Synthesis via the Near-Perfect Absorption of Light. ACS Catalysis.

[98] Li H., Qin F., Yang Z., Cui X., Wang J., Zhangu L. (2017). New Reaction Pathway Induced by Plasmon for Selective Benzyl Alcohol Oxidation on BiOCl Possessing Oxygen Vacancies. J. Am. Chem. Soc..

[99] Ding T., Mertens J., Lombardi A., Scherman O.A., Baumberg J.J. (2017). Light-Directed Tuning of Plasmon Resonances via Plasmon-Induced Polymerization Using Hot Electrons. ACS Photonics.

[100] Wang Y., Wang S., Zhang S., Scherman O.A., Baumberg J.J., Ding T., Xu H. (2018). Plasmon-directed polymerization: Regulating polymer growth with light. Nano Res..

[101] Erzina M., Guselnikova O., Postnikov P., Elashnikov R., Kolska Z., Miliutina E., Svorcik V., Lyutakov O. (2018). Plasmon-Polariton Induced, “From Surface” RAFT Polymerization, as a Way Toward Creation of Grafted Polymer Films with Thickness Precisely Controlled by Self-Limiting Mechanism. Adv. Mater. Interfaces.

[102] Guselnikova O., Marque S.R.A., Tretyakov E., Mares D., Jerabek V., Audran G., Joly J.-P., Trusova M., Švorčik V., Lyutakov O. (2019). Unprecedented Plasmon-Induced Nitroxide-Mediated Polymerization (PI-NMP): A Method for Preparation of Functional Surfaces. J. Mat. Chem. A.

